# Early Detection of Disease Outbreaks

**DOI:** 10.1371/journal.pmed.0020065

**Published:** 2005-02-15

For disease outbreak detection, the public-health community has historically relied on the watchful eyes of doctors and other health-care workers, who have reported individual cases or clusters of cases of particular diseases to health-care and other authorities. The increased availability of electronic health-care data, however, raises the possibility of more automated and earlier outbreak detection and subsequent intervention. Besides diagnoses of known diseases, pre-diagnostic syndromic indicators—such as the primary complaints of patients coming to the emergency room or calling a nurse hotline—are being collected in electronic formats and could be analyzed if suitable methods existed. Martin Kulldorff and colleagues have been developing such methods, and now report a new and very flexible approach for prospective infectious disease outbreak surveillance.

Their method, which they call the “space–time permutation scan statistic,” is an extension of a method called scan statistic. All previously developed scan statistics require either (i) a uniform population at risk (with the same number of expected disease cases in every square kilometer), (ii) a control group (such as emergency visits not due to the disease of interest), or (iii) other data that provide information about the geographical and temporal distribution of the underlying population at risk, such as census numbers. The new method, because of a different probability model, can be used for the early detection of disease outbreaks when only the number of cases is available. It also corrects for missing data and makes minimal assumptions about the spatiotemporal characteristics of an outbreak. To make it widely accessible, the method has been implemented as a feature of the freely available SaTScan software.

**Figure:**
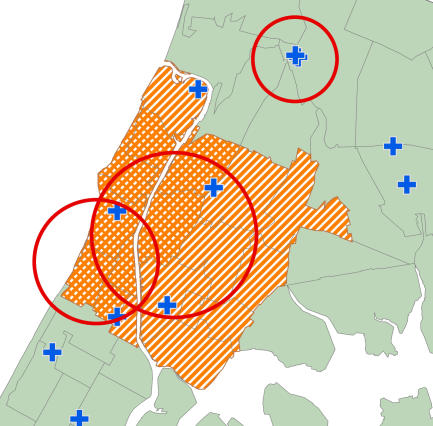
Disease surveillance in New York City

In their article, Kulldorff and colleagues illustrate the utility of the new method by applying it to data collected from hospital emergency departments in New York City. The researchers analyzed diarrhea records from 2002, and did both a “residential analysis” (based on the home address of the patients) and a “hospital analysis” (based on hospital locations). The former has more detailed geographical information, the latter maybe be better able to detect outbreaks not primarily related to place of residence but, for example, school or workplace. They found four highly unusual clusters of diarrhea cases, three of which heralded citywide gastrointestinal outbreaks due to rotavirus and norovirus.

Since November 2003, the space–time permutation scan statistic has been used daily to analyze emergency department data in New York City in parallel with other methods, and it seems to perform well. As the authors discuss, as any other surveillance method, theirs has limitations. Because it adjusts for purely temporal clusters, the method can only detect outbreaks if they start locally (not simultaneously across the entire surveillance area). The less geographically compact an outbreak is, the less power there is to detect it. And some outbreaks, for example, those caused by exposure to an infectious agent in the subway, will be hard to cluster by place of residence or choice of emergency department.

In the present study, Kulldorff and colleagues have applied their method to infectious disease surveillance in a metropolitan area in the United States. As they state, however, “the ability to perform disease surveillance without population-at-risk data is especially important in developing countries, where these data may be hard to obtain.”

